# Bioremediation of Petroleum-Contaminated Soils with Biosurfactant-Producing Degraders Isolated from the Native Desert Soils

**DOI:** 10.3390/microorganisms10112267

**Published:** 2022-11-15

**Authors:** Zheng Li, Ravid Rosenzweig, Fengxian Chen, Ji Qin, Tianyi Li, Jincheng Han, Paula Istvan, Damiana Diaz-Reck, Faina Gelman, Gilboa Arye, Zeev Ronen

**Affiliations:** 1 Zuckerberg Institute for Water Research, Jacob Blaustein Institutes for Desert Research, Ben-Gurion University of the Negev, Sede Boqer Campus, Be’er Sheva 8499000, Israel; lizhen15@msu.edu (L.Z.); qin00128@umn.edu (J.Q.); jincheng@post.bgu.ac.il (J.H.); paula.istvan@gmail.com (P.I.); ddr@bgu.ac.il (D.D.-R.); 2 Geological Survey of Israel, 32 Yeshayahu Leibowitz St., Jerusalem 9692100, Israel; rravid@gsi.gov.il (R.R.); faina@gsi.gov.il (F.G.); 3 French Associates Institute for Agriculture and Biotechnology of Drylands, Jacob Blaustein Institutes for Desert Research, Ben-Gurion University of the Negev, Sede Boqer Campus, Be’er Sheva 8499000, Israel; chenfe@post.bgu.ac.il (F.C.); tianyi0714@gmail.com (T.L.); aryeg@bgu.ac.il (G.A.)

**Keywords:** petroleum contamination, bioremediation, desert soils, hydrocarbon degraders, biosurfactant producers, soil hydrophobicity

## Abstract

A crude oil spill in 2014 resulted in extensive soil contamination of the hyper arid Evrona Nature Reserve in Israel’s Negev Desert. The contaminated soils became highly hydrophobic, threatening the existence of plants in the habitat. We hypothesized that bioaugmenting the soil with indigenous biosurfactant-producing, hydrocarbon-degrading bacteria (HDB) would accelerate the reduction in the soil’s hydrophobicity. We aimed to isolate and characterize biosurfactant-producing HDBs from the desert-contaminated soil and test if they can be used for augmenting the soil. Twelve hydrocarbon-degrading strains were isolated, identified as *Pseudomonas*, and classified as biosurfactants “producing” and “nonproducing”. Inoculating 10^9^ CFU/g of “producing” strains into the polluted soil resulted in a 99.2% reduction in soil hydrophobicity within seven days. At the same time, nonproducing strains reduced hydrophobicity by only 17%, while no change was observed in the untreated control. The microbial community in the inoculated soil was dominated by the introduced strains over 28 days, pointing to their persistence. Rhamnolipid biosynthesis gene *rhl*AB remained persistent in soil inoculated with biosurfactants, indicating in situ production. We propose that the success of the treatment is due to the use of inoculum enriched from the polluted soil.

## 1. Introduction

Oil spills often occur during onshore transportation and storage of crude and refined oil products. These spills pollute the soil and groundwater and can cause severe damage to the environment [1]. Oil pollution during production and transport is a global environmental challenge. An essential feature of oil-contaminated soils is their water repellency compared to uncontaminated soils [2,3]. Hydrocarbons are hydrophobic because they contain many hydrogen–carbon bonds [4]. When oil leaks into the soil, hydrocarbon molecules adsorb onto the soil particles, making the soil hydrophobic [5]. Thus, to a certain extent, soil hydrophobicity indicates oil content in contaminated soil [6]. However, high hydrocarbon degradation does not necessarily imply a significant decrease in soil hydrophobicity [7].

The Evrona Nature Reserve, a hyperarid site in Israel’s Negev Desert, was impacted by two oil pollution events in 1975 and 2014. The contaminated soil from both events was highly hydrophobic. Based on various measurements, both soils were classified as strongly water-repelling, suggesting severe and persistent hydrophobicity [8,9].

Current remediation methods for such contamination include physical, chemical, biological, and integrated remediation. Among them, bioremediation by microorganisms is generally considered environmentally friendly because it is low in energy consumption and does not introduce other pollutants or damage [10]. There are generally two approaches for soil microbial remediation: biostimulation and bioaugmentation. The former refers to the adjustment of environmental parameters such as water, nutrients, and oxygen to improve the activity of the native microbial community. The latter refers to adding bacteria to increase the degradation rate [11,12]. Previous studies showed that a combined biostimulation–bioaugmentation strategy achieved maximum remediation compared to a single remediation method [3,13]. For successful biostimulation and bioaugmentation, two vital prerequisites must be fulfilled: first, improving water penetration in the soil to implement biostimulation, and second, isolating efficient hydrocarbon-degrading bacteria used for bioaugmentation [14].

A laboratory experiment on contaminated soil batches from Evrona monitored soil hydrophobicity and total hydrocarbon concentration for more than 1.5 years while applying a biostimulation approach combined with biosurfactants addition [7]. It was found that although the degradation of total hydrocarbons reached 40~80%, the soil hydrophobicity was still very high [7]. In another study, a greenhouse experiment applied biostimulation and bioaugmentation to the Evrona soils polluted in 1975 and 2014 [6]. They found that bioaugmentation of the 2014 soil with the 1975 sediments, which was assumed to be adapted to oil degradation, was ineffective in reducing soil hydrophobicity. Therefore, the relationship between hydrocarbon content and soil hydrophobicity during the bioremediation process still needs further investigation.

A typical method to increase water penetration in hydrophobic soils is the addition of surfactants [15]. Compared with chemical surfactants, biosurfactants are environmentally friendly and thus became popular in the remediation of oil-contaminated soils [16]. Some oil-degrading bacteria can release extracellular surface-active biosurfactants [17]. Biosurfactants improve water penetration in soil and enhance the hydrocarbons’ bioavailability, which serves as growth substrates [18]. Many studies have reported enhanced hydrocarbon removal by biosurfactants in oil-contaminated soils [7,19,20]. However, in most studies, a biosurfactant produced externally was artificially added to the soil rather than in situ by bacteria [18,21,22]. Inoculation with biosurfactant-producing bacteria could provide nontoxic and biodegradable surfactants at a low cost [23]. Therefore, compared to the simple addition of biosurfactants, it seems a better alternative to isolate from oil-contaminated soil bacteria with hydrocarbon degradation and biosurfactant production functions and reapply them to the soils.

In this study, we propose that isolated indigenous hydrocarbon-degrading and biosurfactant-producing strains from oil-contaminated desert soil would effectively eliminate soil hydrophobicity. The main objective of this study was to evaluate bacteria’s ability to degrade hydrocarbons and reduce soil hydrophobicity. The second objective was to examine the correlations between hydrocarbon contents and soil hydrophobicity.

## 2. Materials and Methods

### 2.1. Enrichment and Isolation of Biosurfactant-Producing Strains

Biosurfactant-producing microorganisms were enriched and isolated from control, and biostimulation experiments [7] of oil-polluted soil from Evrona Nature Reserve. Five grams of soil were inoculated into 100 mL Bushnell Hass Broth (BH, Sigma-Aldrich, St. Louis, MO, USA) mineral medium containing (g/L) 0.2 MgSO_4_, 0.02 CaCl_2_, 1 KH_2_PO4, 1 K_2_HPO4, 1 NH_4_NO_3_, 0.05 FeCl_3_, hexadecane (1% *v*/*v*) as a carbon source and incubated at 25 °C for seven days. Then, 50 µL of the culture broth was transferred into a fresh BH medium and incubated under the same conditions. This procedure was repeated three times to enrich the microbial population. After this, aliquots were spread on BH agar plates (1.5%) with hexadecane (1% *v*/*v*) used as the carbon source. These plates were incubated at 25 °C for seven days. Several colonies that appeared on the agar plates were selected for further purification. These colonies were inoculated into fresh sterile BH agar plates and inoculated into Trypticase soy broth (TSB, DIFCO^®^) agar plates to check their purity. Thirteen single colonies were selected, inoculated into fresh BH mineral medium, and incubated on a shaker at 25 °C for one week.

### 2.2. Characterization of the Isolates by 16S rRNA Sequencing and GEN III MicroPlate

The isolation of pure genomic DNA from the isolated strains was conducted by a commercial kit (GenElute Bacterial Genomic DNA kit, Sigma-Aldrich, St. Louis, MO, USA), according to the manufacturer’s instructions. The concentration of eluted DNA was measured with a NanoDrop Spectrophotometer (ND-1000, Thermo Fisher Scientific, Waltham, MA, USA). The primer set 341f (5′-CCTACGGGAGGCAGCAG-3′) [24] and 907r (5′-CCGTCAATTCMTTTGAGTTT) [25] was used to amplify the 16S rRNA gene of the isolates using PCR. The volume of the reaction mixture was 50 µL, consisting of 25 µL enzyme mix Taq DNA Polymerase (Thermo Fisher Scientific, Waltham, MA, USA), 2 µM forward primer, 2 µM reverse primer, 2 µL DNA template, and 19 µL DNA-free water. The procedure was as follows: initial denaturing at 95 °C for 5 min, 35 cycles consisting of denaturing at 95 °C for 30 s, annealing at 58 °C for 30 s, extension at 72 °C for 20 s, and final extension hold at 72 °C for 5 min. After examining the size of amplified PCR products on 1.5% agarose gel, sequencing of the PCR product was performed on an Applied Biosystems 3500xL Genetic Analyzer (Applied Biosystems, Waltham, MA, USA) at the Genomic unit of the Ben Gurion University of the Negev. The sequences were deposited in the gene bank with accession numbers MW857535–MW857545.

Phenotypic and physiological characterization of the isolates was performed with the GEN III MicroPlate (Biolog, Hayward, CA, USA). The isolates were grown on the R2A agar plates, inoculated in the microplate according to the manufacturer’s instructions, and then incubated at 30 °C. After 24 h, the OD of each well was measured at 600 nm in an Infinite M200 microplate reader (Tecan, Männedorf, Switzerland). The negative control OD normalized each well’s OD data in the microplates. Based on the normalized OD values, a principal component analysis (PCA) plot was generated by the web tool ClustVis [26] to compare the numerical response and to distinguish the close strains based on their physiological characteristics and substrate utilization patterns.

### 2.3. Screening of Biosurfactant Production Activity

A loop-full of a single colony grown on the BH agar plates with hexadecane (1% *v*/*v*) was inoculated in flasks of sterilized BH medium with 1% hexadecane and incubated in a shaker at 200 rpm and 30 °C for one week. At the end of the one-week incubation, the cells in the culture broth were removed by centrifuge (Heraeus, Hanau, Germany) at 6000 rpm and 4 °C for 15 min. Then, the supernatant was filtered through PVDF 0.45 µm filters (Merck Millipore Ltd., Carrigtwohill, Ireland). The cell-free culture solutions were used for the following screening tests: oil-spreading assay, emulsification index (E24) assay, and surface tension measurements at the liquid–air interface.

The oil-spreading experiment is a modified version of the method described in a previous study [27]. Briefly, 10 µL crude oil, obtained from Tzuk Tamrur 3 borehole near Arad, was added to the surface of 20 µL distilled water in a Petri dish, followed by 10 µL cell-free culture broth. The diameter of the oil-free clear zone is a measure of the oil displacement activity. A result was considered positive for biosurfactant production when the diameter of the transparent area was at least 5 mm [20]. As the crude oil we used was too dense and the spreading activity was affected by many factors, such as the temperature and the time that the oil was in contact with the water surface, the results of the oil-spreading assay were not comparable between replicates. Therefore, the oil-spreading assay was used as a qualitative method to justify the biosurfactant production activity.

The measurement of emulsification activity was described earlier [28]. Two milliliters of hexadecane and 2 mL cell-free culture broth were vortexed at the maximal speed for 2 min and then kept static for 24 h. Emulsification was then characterized by the E24 index, which is the ratio of the emulsion layer’s height to the fluid’s total height.

Surface tension measurement at the liquid–air interface is a standard method to screen and quantify biosurfactant production [29]. The surface tension measurement of the cell-free broth mentioned above was conducted by the Wilhelmy plate method in a force tensiometer (DCAT15, Data physics Co., Filderstadt, Germany). Fifteen milliliters of the culture were equilibrated in a small weighing dish to measure the surface tension. The surface tension measurement was conducted three times for the same culture sample.

The cetyl trimethyl ammonium bromide (CTAB)–methylene blue agar plate assay was conducted based on the technique proposed by Joy et al. [30]. Mineral salt medium (SW) was prepared using the following chemicals (per liter): 20 g glycerol, 0.7 g KH_2_PO_4_, 0.9 g Na_2_HPO_4_, 2 g NaNO_3_, 0.4 g MgSO_4_·H_2_O, 0.1 g CaCl_2_·2H_2_O, and 2 mL of a trace solution containing 2 g FeSO_4_·7H_2_O, 1.5 g MnSO_4_·H_2_O, and 0.6 g (NH_4_)_6_Mo_7_O_24_·4H_2_O. CTAB–methylene blue agar plates were prepared by adding 0.2 g CTAB, 0.005 g methylene blue, and 15 g agar to the above medium. The isolates were grown in the liquid medium with glycerol as a carbon source in shaker flasks at 34 °C for 24 h. It was followed by spreading 10 µL culture broth on the CTAB–methylene blue agar plates. The plates were then incubated at 34 °C for 24 h and later kept at 4 °C for an additional 48 h. A biosurfactant-producing culture of *Pseudomonas aeruginosa* PAO1 (provided by Prof. Moshe Herzberg, Ben Gurion University, Israel) served as a positive control [31]. Blue halos formed around the biosurfactant-producing colonies indicate positive results for biosurfactant production [32]. The diameters of the blue-halo areas were measured to estimate the production of anionic glycolipid biosurfactant.

Liquid mineral salt medium with glycerol as the carbon source was prepared as described above and used to culture the isolates at 30 °C for three days. The same procedure was conducted to obtain the cell-free culture broth, and measure the surface tension. This measurement aimed to compare biosurfactant production when hexadecane is used as a carbon source to their production when glycerol is used instead.

### 2.4. Preparation of Inoculum and Soil Batch Incubation

We used two representative oil-degrading bacteria strains for the soil incubation experiment, NS1 and CT9. The strains were cultivated in sterile 250 mL Erlenmeyer flasks containing 100 mL Luria Broth (Difco) growth medium until log phase (~10 h at 30 °C, on an orbital shaker at 150 rpm). The bacterial culture was harvested by centrifugation (5000× *g* for 10 min at 4 °C). The supernatant was removed, and the cells were resuspended in 40 mL of sterilized nutrient solution (3 g/L ammonium sulfate and 150 mg/L potassium phosphate were dissolved in tap water). The washing process was repeated three times. The final bacterial suspension was diluted with sterilized nutrient solution to ~2.6 × 10^10^ CFU mL^−1^; this was the standard suspension used for the soil incubation experiments unless noted otherwise. The concentrations of three strains were determined by dyeing the culture with the BacLight LIVE/DEAD Bacterial Viability Kit (Molecular Probes, Eugene, OR, USA) and observation under a fluorescence microscope (Olympus, Japan) using a 40× magnification objective.

### 2.5. Bioaugmentation Experiment

The oil-polluted soil [7] was sieved through a 2 mm sieve. Forty-five grams of soil were added into a plastic box, and 1.74 mL of bacteria suspension was added into the soil and thoroughly mixed to reach 20% saturation and a target bacteria concentration of ~10^9^ CFU g^−1^. The three soil treatments included control soils (without inoculation), soil inoculated with NS1 (hereafter called NS1), and soil inoculated with CT9 (hereafter called CT9). Each treatment comprised three biological replicates. The plastic boxes were sealed with covers and placed in the dark at 25 °C. The boxes were mixed by shaking for 10 s every two days. Soil samples (9 g) were collected once a week from each box, with the total incubation time being one month. A small amount of sterilized tap water was added during each sampling to ensure 20% water saturation.

### 2.6. DNA Extraction, Real-Time Polymerase Chain Reaction (qPCR), Illumina Sequencing, and Microbial Community Analyses

#### 2.6.1. DNA Extraction and qPCR

Soil samples (0.25 g) from three soil treatments were collected on day 0, day 14, and day 28 during the 28-day incubation. The microbial DNA was extracted using the DNeasy PowerLyzer PowerSoil Kit (Qiagen, Hilden, Germany), following the manufacturer’s instructions. The DNA extracts were used to quantify the genes *alkB* and *rhlAB*. The amplification of gene *alk*B was performed using the primer set Alk-BFB (5′-GGTACGGSCAYTTCTACRTCGA-3′) and Alk-BRB (5′-CGGRTTCGCGTGRTGRT-3′) [33], and the amplification of gene *rhl*AB was by Rhlabf (5′-CAGGCCGATGAAGGGAAATA-3′) and Rhlabr (5′AGGACGACGAGGTGGAAATC-3′) [34].

The reaction mixture (20 µL) contains a 10 µL enzyme mix (qPCR-BIO SyGreen Blue Mix Lo-Box, PCR Biosystems Inc., Wayne, PA, USA), 0.4 µM forward primer, 0.4 µM reverse primer, 1 µL DNA template, and 8.2 µL nuclease-free water. Real-time PCR was conducted by a CFX96 Touch™ Real-Time PCR Detection Systems (Bio-Rad, Hercules, CA, USA). The amplification cycle was as follows: initial denaturation at 94 °C for the 30 s, followed by 40 cycles of denaturation at 94 °C for 5 s, annealing at 64 °C for 15 s, extension at 72 °C for 10 s; and then hold at 55 °C for 30 s. The amplification products were run using 1.5% agarose gel electrophoresis to check for primer dimers and nonspecific bands.

#### 2.6.2. Amplicon Sequencing

The V3-V4 region of the 16S rRNA gene was amplified by polymerase chain reaction (PCR) using the primers CS1-341F (5′-CCTACGGGAGGCAGCAG-3′) and CS2-806R (5′-GGACTACHVGGGTWTCTAAT-3′) [19]. The PCR products were submitted to the University of Illinois at Chicago Core for Research Informatics (UICCRI) for amplicon sequencing [35]. The raw sequences in the format of fastq were deposited at sequencing read archives (SRA) of the National Center Biotechnology Information (NCBI) under the BioProject PRJNA811126 (accession numbers from SAMN26309639 to SAMN26309665).

#### 2.6.3. Bioinformatics

The fastq format files of the amplicon sequencing data were analyzed by Mothur (version 1.44.2) [36]. Following the trimming of the raw sequences and alignment against the SILVA bacteria database (Release 138), the chimeras, mitochondrial, and chloroplast lineage sequences were removed. Then, the operational taxonomic units (OTU) were classified at the 0.03 cutoff, and the downstream analysis was performed in R (version 4.1.1) with Rstudio (version 1.0.5042) (RStudio_Team, 2020). The R packages file2meco (version 0.2.0) ([37], phyloseq (version 1.36.0) [38], and microbiome (version 1.14.0) [39] were used to generate bar plots at the phylum, and perform the principal coordinate analysis (PCoA) and the alpha diversity analysis (Chao1, Shannon’s values, Simpson).

### 2.7. Soil Hydrophobicity and Hydrocarbon Analysis

The soil samples (9 g) were oven-dried at 65 °C overnight and left in the open air for one day. The soils were placed in 50 mL centrifuge tube caps to identify the change in soil hydrophobicity through time. The water-drop penetration time (WDPT) method [40] was used to determine the degree of soil hydrophobicity. Briefly, a 50 µL droplet of water was placed on the soil surface, and the time required for the water drop to penetrate the soil surface was measured. If the WDPT value was higher than 3600 s, a value of 4000 s was arbitrarily assigned. This analysis was conducted in triplicates.

The petroleum hydrocarbons were extracted and analyzed by a gas chromatography–mass spectrometry (GC–MS) system (6890 N-5975, Agilent, Santa Clara, CA, USA). Our previous paper described the details of the hydrocarbon extraction and GC–MS analysis procedure [7].

## 3. Results

### 3.1. Biosurfactants Screening

**Figure 1:**
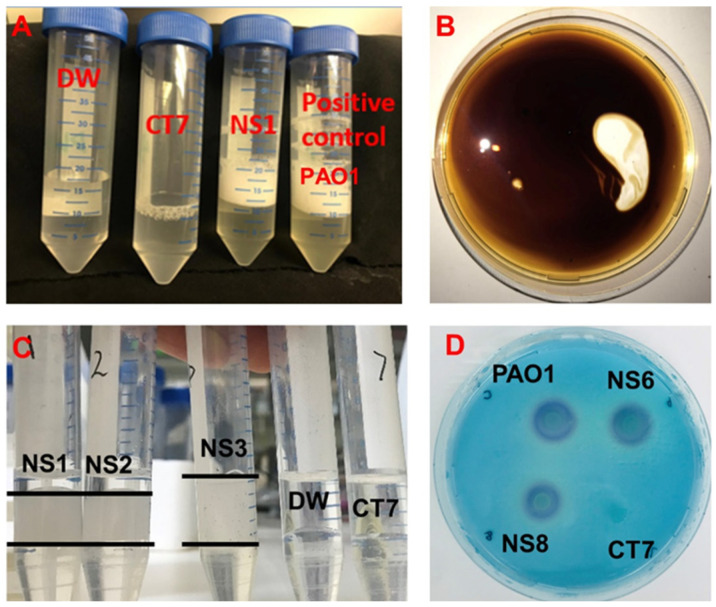
Biosurfactant detection by the isolated strains. (**A**) The initial screen of the strains in BH culture broth; potential biosurfactant-producing strains produced foams. (**B**) Positive results in the oil spread test; 10 µL cell-free culture broth was added onto the crude oil layer on the surface of the water. When the diameter of the clear zoon is more than 5 mm, the oil displacement activity is positive. (**C**) Emulsified height in the emulsification test. (**D**) CTAB agar plates with four well, each added with 10 µL culture medium. The blue halo area indicated the production of anionic glycolipid biosurfactant by the colonies.

Potential biosurfactant-producing strains produced foams in the culture broth in the presence of hexadecane (Figure 1A). The characteristics of the oil-spreading activity assay, the cetyl trimethyl ammonium bromide–methylene agar test, the emulsification activity assay, and the surface tension reduction (compared to pure water) for these strains are summarized in Table 1. In this study, the oil-spreading assay was used as a qualitative measure to check the production of biosurfactants in the cell-free broth. The strains that presented a positive oil-spreading activity (Figure 1B) were denoted as NS strains (NS1, NS2, NS3, NS4, NS5, NS6, and NS8), and strains that were shown to be negative in the oil-spreading assay were denoted as CT strains (CT7, CT9, CT10, CT11, CT12, and CT14). The results of the emulsification activity agreed with those of the oil-spreading assay. As shown in Table 1, the surface tension measurements of the BH cell-free broth with hexadecane as a carbon source displayed a reduction from ~68 to ~43 mN/m in the surface tension of the NS strains and from ~68 to ~40 mN/m in that of PAO1.

In contrast, a reduction from ~68 to 55 mN/m in the surface tension of the CT strains was observed. The SW cell-free culture broth with glycerol as a carbon source reduced the surface tension from ~68 to ~28 mN/m in the NS strains and from ~68 to ~29 mN/m in the PAO1 strain. These strains also presented emulsification activity in the culture broth (Figure 1C). Further, the biosurfactant production potential of the NS strains was confirmed by the CTAB method, where the seven NS strains were found to be positive, with the area of the blue halo ranging from ~178 to ~227 mm^2^, whereas the size the halo of the PAO1 strain was ~227 mm^2^ (Figure 1D and Table 1). The CT strains were observed to be negative in the CTAB method.

### 3.2. Isolates Characterization

Thirteen strains were isolated from the microcosm with oil-contaminated soils in Evrona Nature Reserve. The 16S rRNA sequences of the isolates were aligned to the reference sequences found from BLAST. Based on the sequence analysis, all the isolates were identified as members of the *Pseudomonas* genus. The phenotypic characteristics of the isolates were typical to this genus, with 61 ± 11 carbon sources utilized after 24 h incubation in the Biolog plate (Table S1). The strains showed extensive antibiotic resistance; however, most of them were sensitive to Aztreonam. Most of the strains tolerated 4% NaCl but not 8% NaCl, and were able to grow at pH 6.

**Figure 2:**
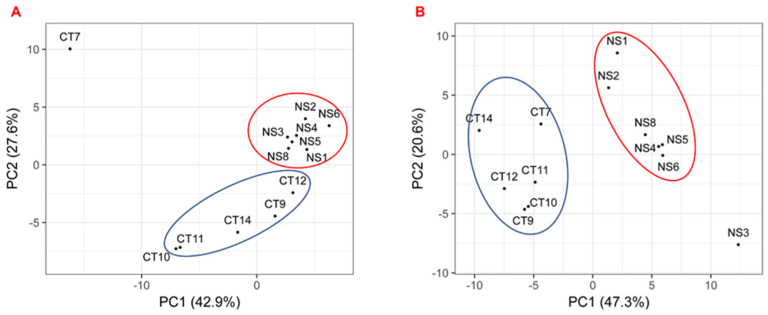
Principal component analysis (PCA) of all 13 strains (**A**) after 24 h incubation; (**B**) after 48 h incubation. The PCA plots, generated by the normalized OD values, represent isolated strains characterized by the Biolog by two or more axes: PC1 (42.9%) and PC2 (27.6%) after 24 h incubation; PC1 (47.3%) and PC2 (20.6%) after 24 h incubation.

The PCA analysis based on the substrate utilization pattern of the Biolog assay shows that the NS strains having biosurfactant production activity were clustered close to each other, and the CT strains without biosurfactant production potential generated a second cluster (Figure 2). As a result, NS1 and CT9 were selected as the representatives for the biosurfactant-producing and nonbiosurfactant-producing strains, respectively, to be used in subsequent bioremediation incubation experiments.

### 3.3. Bioaugmentation Experiment

**Figure 3:**
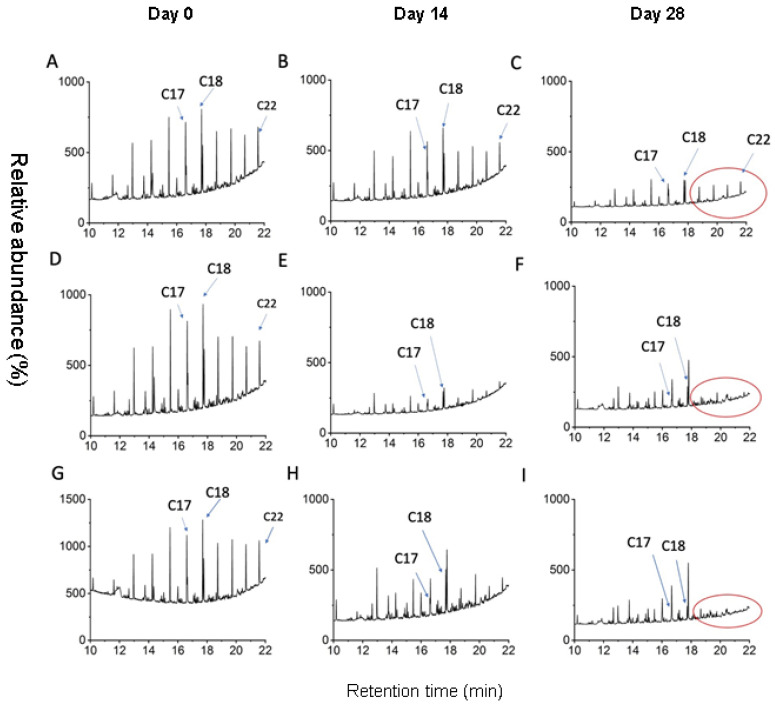
Gas chromatography–mass spectrometry (GC–MS) chromatograms of hydrocarbons extracted from the control soils (**A**–**C**), soils bioaugmented with P. strains CT9 (**D**–**F**), and soils treated with P. strains NS1 (**G**–**I**) at the initial condition, after 14-day incubation, and after 28-day incubation.

During the 28-day incubation period of the bioaugmentation experiment, individual hydrocarbons in the soils were removed to different degrees (Figure 3). The hydrocarbons in the soils augmented with CT9 or NS1 degraded rapidly in the first 14 days. The control soils in this study were treated with 20% water saturation and nutrients (biostimulation). Therefore, it was not surprising that hydrocarbon degradation occurred during incubation of the soil. However, after 28 days, the soil was still enriched with longer-chain alkanes (>C18) (Figure 3C). In contrast, the abundance of longer-chain alkanes was very low for the soils bioaugmented with the CT9 or NS1 strains at the end of incubation (Figure 3F,I).

**Figure 4:**
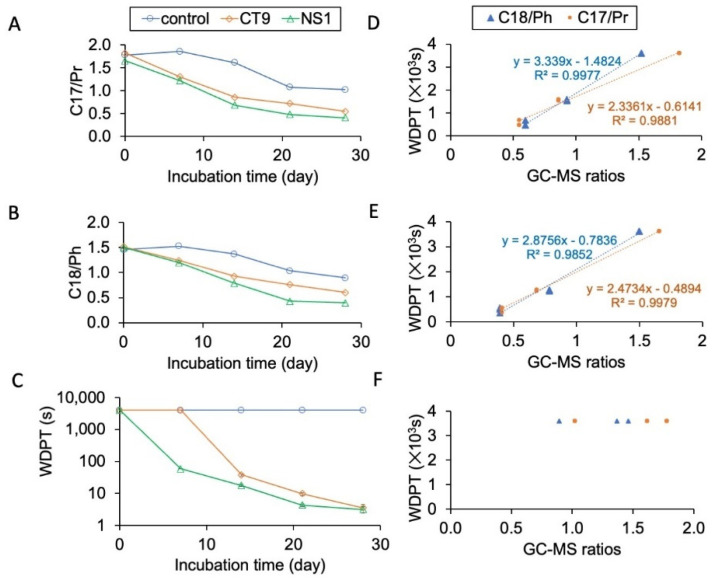
Changes in relative abundance ratios of (**A**) n-C17/pristane (n-C17/Pr) and (**B**) n-C18/phytane (n-C18/Ph) and (**C**). The correlation of ratios with log water-drop penetration time for NS1 (**D**), CT9 (**E**), and control (**F**) treatments during the 28-day incubation.

The ratios of C17-alkane/pristine (C17/Pr) and C18-alkane/phytane (C18/Ph) decreased during the incubation for all three soils (Figure 4A,B). The reduction rates were the highest in the NS1-inoculated soils, followed by the CT9-inoculated soils, and the lowest in the control soils. The results indicated that the soil was still hydrophobic after the incubation (Figure 4C). The ratios were significantly correlated with the log WDTP for NS1 and CT9 treatments (Figure 4D,E) but not for the control (Figure 4F). It was not expected that the WDPTs of the CT9 and NS1 soil treatments would decrease by 100-fold to less than 10 s at the end of the incubation. It was also interesting to note that the decrease in WDPT in the CT9 treatment lagged for seven days while the values for the NS1 treatment decreased from the beginning. Yet, the final level of the WDPT in the CT9 and NS1 soil treatments was at the same level.

#### 3.3.1. Microbial Community Change

**Figure 5:**
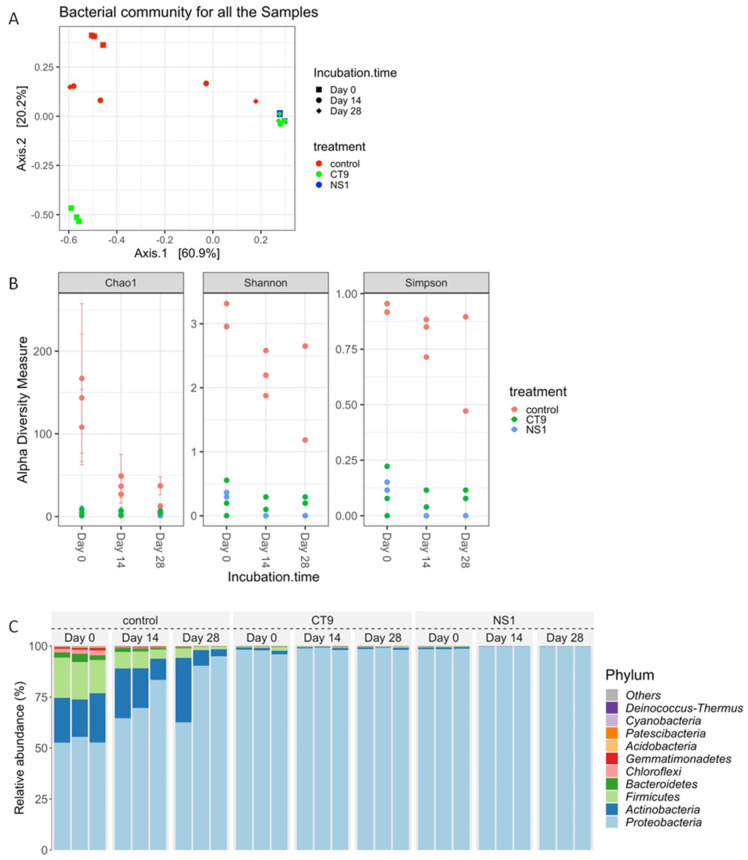
(**A**) PCoA (**B**) alpha diversity indices (**C**) most abundant phyla in the three soil treatments during the 28-day incubation.

**Figure 6:**
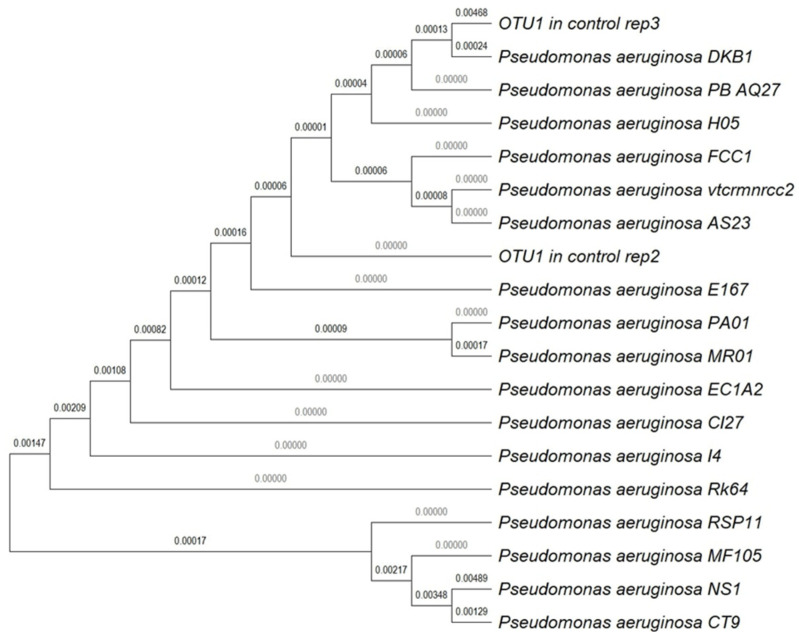
Phylogenetic tree built by the neighbor-joining method by MEGA11.

The microbial communities of NS1, CT9, and control treatments were evaluated. The relative abundance of the different phyle was examined. As expected, the bacterial communities of the control soils differed from the CT9 and NS1 treatments (Figure 5A). The alpha diversity indices were much higher in the control soils than in the other two soils, and the values of diversity decreased between day 0 and 14 for the control soils (Figure 5B). As shown in Figure 5C, *Proteobacteria* was the most dominant phylum in all the soil treatments. In addition, the abundance of *Proteobacteria* increased while *Firmicutes*, *Bacteroidetes*, and *Chloroflexi* became less abundant during the 28-day incubation period in the control soil treatments. The phylogenetic tree constructed by the neighbor-joining method of the partial 16S rRNA gene showed that at the end of the incubation, the most abundant strains in the control soils were closely related to the species of the genus *Pseudomonas*, which also includes the isolates NS1 and CT9 (Figure 6).

We did not re-isolate the inoculated strains from the treated soils in the current study. The microbiome data (Figure 5C) do not provide information on the abundance at the species level. Thus, there is a possibility that the effects observed are due to horizontal gene transfer from the augmented strains to the native community. For example, TOL plasmid coding for m-toluate degradation was transferred from the augmented host *Pseudomonas fluorescens* strain OS81 to the native soil community during three months of soil incubation [41]. Other studies found that environmental parameters such as temperature, water content, pollutants concentrations, and presence of plants affected the frequency of horizontal transfer of TOL-like plasmids encoding catechol 2,3-dioxygenase (C23O) in soil [42]. Because we do not know if hydrocarbon degradation and biosurfactant production are associated with the mobile genetic elements, we should further explore the genome of introduced strain (CT9 and NS1) to determine this possibility.

#### 3.3.2. Functional Gene Abundance

**Figure 7:**
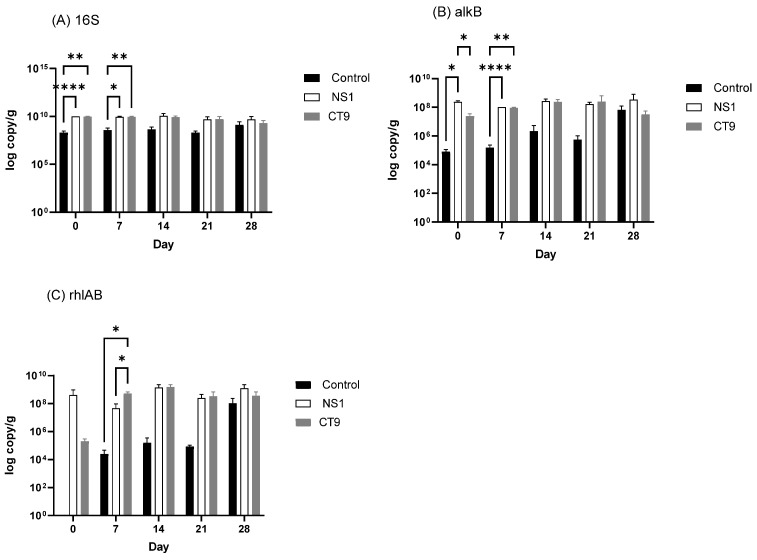
Gene abundance of (**A**) 16S rRNA, (**B**) *alk*B, and (**C**) *rhl*AB quantified by real-time PCR in the NS1, CT9, and control soils during the 28-day incubation. Note: the numbers are mean values of triplicates. One-way ANOVA conducted the statistical test. “****” indicates *p* < 0.0001, “**” indicates *p* < 0.01, and “*” indicates *p* < 0.05.

The copy number of the 16S rRNA gene in the control was 1% of the NS1 and CT9 inoculated soils. It increased by an order of magnitude but was still lower than the NS1 and CT9 treatments (Figure 7A). The 16S rRNA gene copy numbers in the augmented soils were stable. The abundance of the alkane hydroxylase gene (*alk*B) and the rhamnosyltransferase gene complex (*rhl*AB) were detected during the 28-day incubation (Figure 7B, C). At the beginning of the incubation, the abundance of *alk*B was the highest in the NS1 treatment, followed by the CT9 treatment, and then in the control soil (Figure 7B). After 14 days, there was no notable difference in the *alk*B abundance between the NS1 and CT9 soils, while this quantity was lower in the control soils than in the other two soils. Two-way ANOVA indicates a significant (*p* = 0.046) effect of the treatment on *alkB* abundance. The *rhl*AB gene was below the detection limit in the control at the beginning of incubation, while it was detected in the NS1 and CT9 soils (Figure 7C). After 7-day incubation, the abundance of *rhl*AB was significantly higher in the CT9 soils than in the control soils. After 14-day incubation, the abundance of *rhlAB* gene was markedly higher in the NS1 soils than in the control soils but not higher than its abundance in the CT9 treatment. At the end of the one-month incubation period, the abundance of *alk*B and *rhl*AB genes was not significantly different among the NS1, CT9, and control treatments.

## 4. Discussion

This study characterized biosurfactant-producing strains isolated from oil-contaminated desert soil and their ability to reduce soil hydrophobicity. We assumed that hydrocarbon-degrading bacteria from the polluted soil would produce biosurfactants as a strategy for accelerating biodegradation. To our knowledge, the reduction in soil hydrophobicity by inoculating native biosurfactant-producing hydrocarbon degraders in arid soil systems has not yet been reported.

Based on the sequence analysis, all the NS and CT isolates were identified as members of the *Pseudomonas* genus. The NS and CT isolates had a different response in the Biolog plate assay. The separation between the populations may be due to rapid surfactant production by the NS isolates.

Various screening methods were applied to assess biosurfactant productivity and surface activity from the different isolates. The NS strains which presented positive activity in the oil-spreading assay were also positive in the emulsification assay, with the E24 index ranging from 42.4% to 51.9%. These results agree with other studies. For instance, a marine biosurfactant-producing, oil-degrading bacteria displayed high emulsification activity (>30%) [43]. In another study, a biosurfactant-producing strain, *Shewanella* sp. BS4 isolated from oil-contaminated soil, yielded 62.4% and 11.4% E24 values for cell and cell-free broth, respectively [44].

The CTAB assay is a specific method for detecting anionic biosurfactant production based on forming an insoluble ion pair with various cationic substances [32]. Therefore, the use of the CTAB–methylene blue plates confirmed the production of anionic biosurfactant by strains NS1, NS2, NS3, NS4, NS5, NS6, and NS8.

Biosurfactants such as rhamnolipids can lower water surface tension and increase the solubility of organic compounds, thus increasing their bioavailability and enhancing hydrocarbon removal [45]. Efficient surfactants can lower the surface tension of water from 72 to 35 mN/m [46]. The NS isolates reduced the surface tension from ~68 to 28 mN/m using glycerol and from ~68 to 43 mN/m using hexadecane as a carbon source. Our results agree with a previous study, which showed that rhamnolipids-producing strain *Pseudomonas* aeruginosa DAUPE 614 grew on glycerol and reduced the surface tension to 27.3 mN/m [44]. Our observations confirm that the NS isolates produce biosurfactants. Perfumo et al. [33] found that the carbon source affected both biosurfactant quantity and structures. The variety of rhamnolipids can dramatically decrease when produced on hexadecane compared to water-soluble glycerol [32]. The report may explain why the SW cell-free broth with glycerol as a carbon source yielded ~59% surface tension reduction relative to a negative control, while the BH broth with hexadecane as a carbon source yielded only ~37% reduction for the same strains.

The application of biosurfactant-producing consortia in hydrocarbon bioremediation was examined in different habitats [18,23,47,48]. For example, *Pseudomonas aeruginosa* S5 isolated from coking wastewater achieved 55% PAHs removal in the coking sludge [49]. *Pseudomonas aeruginosa* SR17 can produce rhamnolipids and remove TPH by up to 80% [18]. In the current study, two strains (NS1 and CT9) were inoculated into the oil-contaminated soils to explore the bioremediation potential of the native biosurfactant-producing hydrocarbon degraders. Rapid degradation of hydrocarbons and decline in soil hydrophobicity (Figure 3 and Figure 4) suggest that the two isolates survived and adapted well to the native desert soil environment. This was also supported by the enrichment of genes *alkB* and *rhlAB* in all three soils at the end of the one-month incubation period.

It was hypothesized that the non-biosurfactant-producing strain CT9 would be less effective in removing the hydrocarbons. After a 7-day lag time, the soil hydrophobicity of the CT9 treatment decreased almost as fast as in the NS1 treatment (Figure 4). The biosurfactant functional gene *rhlAB* was also detected in the CT9 soils during the incubation. It is suggested that CT9 can also produce biosurfactants after sufficient incubation time and degrade hydrocarbons. *Pseudomonas* bacteria have been extensively explored for their potential to degrade hydrocarbons and produce biosurfactants [49,50,51,52,53]. Specifically, *Pseudomonas* was one major isolated alkane-degrading group from nutrient-amended, oil-contaminated desert soils [54]. Further, in the current study, Pseudomonas (e.g., NS1 and CT9) was the major biosurfactant-producing group isolated from the oil-contaminated soils and reduced biodegradation markers (C17/Pr and C18/Ph) by ~60% and ~70%, respectively. While WDPT was correlated with the degradation markers in the augmented soil, it was not apparent in the control. Thus, the results suggest concurrent biosurfactant production and hydrocarbon degradation are needed to reduce soil hydrophobicity (Figure 4D–F). The results agree with our earlier observations where external biosurfactants addition reduced soil water, repealing [7]. At the end of the 28-day incubation, the most dominant strain in the control soils was also classified to belong to the same genus (*Pseudomonas*) as the CT9 and NS1 strains (Figure 5 and Figure 6). We are proposing that the inoculated strains were the ones that were functioning in the native hydrocarbon degradation. After long-term incubation, Gamaproteobacteria taxa such as *Pseudomonas* may constitute the majority in soil microbial communities, because they adapted to rapid hydrocarbon degradation. *Pseudomonas* take part in the biodegradation of petroleum products in almost all hydrocarbon-contaminated areas, including arid soils.

Earlier studies demonstrated that a hydrocarbon-degrading consortium was found to present better diesel degradation in the presence of biosurfactant-producing strains than the biosurfactant itself [23]. Based on our research, exogenously added biosurfactants achieved 45% total petroleum hydrocarbon (TPH) removal in the oil-contaminated soil [7]. In addition, the soil hydrophobicity changed from severely non-wettable to strongly non-wettable after 1.5-year incubation. In the current work, even though the TPHs were not monitored, the soils inoculated with NS1 and CT9 changed from severely non-wettable to wettable in one month. It is conclusive evidence that biosurfactant-producing HDBs are more efficient in improving hydrocarbon degradation and reducing the induced soil hydrophobicity than the previous biostimulation combined with the addition of biosurfactants [7].

Hydrocarbon molecules interfere in the interaction between soil particles and water by forming thin films over the soil particles [55] and changing soil surface properties by increasing soil hydrophobicity [8]. In our previous study, TPH concentration was positively correlated with soil hydrophobicity characterized by the molarity of the ethanol droplet method in the oil-contaminated desert soils [7]. A linear relationship was found between soil hydrophobicity and TPH at low concentrations (<2000 mg/kg) in both sandy and clayey soils [56]. In another study, soil hydrophobicity increased with increasing concentrations of crude oil in clayey alluvial soil [52]. These agree with our current findings, which indicate that the soil hydrophobicity was linearly correlated with the hydrocarbon degradation indicators. Therefore, the severity of soil hydrophobicity was related to the concentrations of hydrocarbons within the soils. Moreover, heavier crude oil with more long-chained hydrocarbons presented more server soil hydrophobicity than lighter oils at the same concentrations [55]. Therefore, the long-chained heavy hydrocarbon residues may be responsible for the current study’s extreme hydrophobicity in untreated (control) oil-contaminated soils.

A hyperarid site in Israel’s Negev Desert, Evrona Nature Reserve, was affected by two oil pollution events in 1975 and 2014. Both events produced highly hydrophobic soil. The soils were classified as strongly hydrophobic according to various measurements [8,9], indicating a persistent hydrophobicity that prevents water penetration and ecosystem recovery. Our finding presents a novel approach where bioaugmentation with indigenous hydrocarbon-degrading, biosurfactant-producing bacteria reduce rapidly the water repellency of the soil and, by that, accelerates the potential for the desert soil habitat recovery.

## 5. Conclusions

In this study, we isolated biosurfactant-producing hydrocarbon degraders and investigated hydrocarbon bioremediation potential by inoculating the isolates in the oil-contaminated soils. The two selected strains, NS1 and CT9, survived and adapted to the desert soils with 20% water saturation. Surprisingly, the biosurfactant “producing” strain NS1 and the “nonproducing” strain CT9 improved hydrocarbon removal and soil hydrophobicity to the same level, even though CT9 showed a 7-day lag. *Pseudomona*s became prevalent in the control soils after 28-day incubation, which further suggested that the isolated strains function in the native soils. The gene *rhl*AB coding for rhamnolipids biosynthesis was persistent in the inoculated soil, pointing to in situ biosurfactant productions. Finally, our results suggest that the potential using biosurfactant-producing hydrocarbon degraders in desert soil systems enhances in situ hydrocarbon contamination removal and soil hydrophobicity mitigation. This approach should be further examined under more realistic field conditions.

## Figures and Tables

**Table 1:** Biosurfactant-activity screening assay.

Isolate Code	Oil Spreading	E_24_ Index	Surface Tension (mN/m) ^a^	Surface Tension (mN/m) ^b^	CTAB Halo Area (mm^2^)
NS1	+	48.2% ± 0.5% (+)	41.86 ± 0.03 (++)	27.70 ± 0.04 (++++)	178 ± 15.59
NS2	+	44.4% ± 0.9% (+)	43.70 ± 0.04 (++)	28.92 ± 0.04 (++++)	186.83 ± 7.94
NS3	+	51.9% ± 0.4% (++)	44.11 ± 0.07 (++)	27.56 ± 0.08 (++++)	196.17 ± 14.00
NS4	+	42.6% ± 0.3% (+)	44.50 ± 0.04 (++)	27.63 ± 0.03 (++++)	200.75 ± 8.23
NS5	+	48.2% ± 0.5% (+)	43.19 ± 0.08 (++)	27.67 ± 0.04 (++++)	186.83 ± 7.94
NS6	+	48.2% ± 0.7% (+)	44.10 ± 0.03 (++)	28.82 ± 0.03 (++++)	205.5 ± 8.23
NS8	+	51.9% ± 0.2% (++)	41.81 ± 0.05 (++)	27.65 ± 0.02 (++++)	196.94 ± 1.62
CT7	-	-	56.40 ± 0.09 (+)	/	/
CT9	-	-	56.76 ± 0.02 (+)	/	/
CT10	-	-	55.51 ± 0.03 (+)	/	/
CT11	-	-	56.15 ± 0.04 (+)	/	/
CT12	-	-	55.39 ± 0.07 (+)	/	/
CT14	-	-	54.22 ± 0.05′ (+)	/	/
PAO1	+	63% ± 0.9% (+++)	40.03 ± 0.05 (++)	29.61 ± 0.03 (++++)	227.1 ± 3.49
Neg	-	-	68.13 ± 0.03 (-)	64.06 ± 0.07 (-)	

^a^—The experiment was conducted with BH cell-free culture broth with hexadecane as a carbon source. ^b^—The experiment was conducted with SW medium cul cell-free culture broth with glycerol as a carbon source. /—The experiment was not conducted; PAO1—Biosurfactant-producing *Pseudomonas aeruginosa* PAO1; Neg—The result of distilled water used as a negative control. Oil spreading: “+”—positive oil-spreading activity; “-”—no oil-spreading activity. E24 index: “+++”—E24 > 60%; “++”—E24 = 50–60%; “+”—E24 = 40–50%; “-”—no emulsification activity. Surface tension: “++++” surface tension < 30 mN/m; “+++” surface tension 30–40 mN/m; “++” surface tension 40–50 mN/m; “+” surface tension 50–60 mN/m; “-”—no surfactant activity.

## Data Availability

The data presented in this study are available on request from the corresponding author.

## Supplementary Materials

The following supporting information can be downloaded at: https://www.mdpi.com/2076-2607/10/11/2267/s1, Table S1: Biochemical analysis of isolates by GEN III Micro Plate after (a) 24 h incubation and (b) 48 h incubation.
