# Supplementary material for: Bioremediation of Petroleum-Contaminated Soils with Biosurfactant-Producing Degraders Isolated from the Native Desert Soils
Source: Microorganisms. 2022 Nov 15;10(11):2267. doi: 10.3390/microorganisms10112267 (PMC9694877; doi:10.3390/microorganisms10112267)
Supplement: Supplementary file 1 [file microorganisms-10-02267-s001.zip › microorganisms-2015915-supplementary.pdf]

microorganisms

MDPI

Article

Bioremediation of Petroleum-Contaminated Soils with Biosurfactant-Producing Degraders Isolated from the Native Desert Soils

Li Zheng [1,2], Ravid Rosenzweig [2], Chen Fengxian [3], Ji Qin [1], Tianyi Li [3], Han Jincheng [1], Paula Istvan [1], Damiana Diaz-Reck [1], Faina Gelman [2], Gilboa Arye [3] and Zeev Ronen [1,*]

[1] Zuckerberg Institute for Water Research, Jacob Blaustein Institutes for Desert Research, Ben-Gurion University of the Negev, Sede Boqer Campus, Be'er Sheva 8499000, Israel
[2] Geological Survey of Israel, 32 Yeshayahu Leibowitz St., Jerusalem 9692100, Israel
[3] French Associates Institute for Agriculture and Biotechnology of Drylands, Jacob Blaustein Institutes for Desert Research, Ben-Gurion University of the Negev, Sede Boqer Campus, Be''er Sheva 8499000, Israel
* Correspondence: zeevrone@bgu.ac.il

Supplementary information

Table S1. Biochemical analysis of isolates by GEN III MicroPlate after (a)24-h incubation and (b) 48-h incubation.

(a)

	NS1	NS2	NS3	NS4	NS5	NS6	NS8	CT7	CT9	CT10	CT11	CT12	CT14
Dextrin	-	-	+	-	+	-	-	+	+	+	+	+	+
D-Maltose	-	-	+	-	+	-	+	+	+	+	+	+	+
D-Trehalose	+	-	+	+	+	-	+	+	-	+	+	-	+
D-Cellobiose	-	-	+	-	+	-	-	+	-	+	+	-	+
Gentiobiose	-	-	+	-	+	-	+	+	-	+	+	-	+
Sucrose	-	-	-	-	+	-	+	+	-	+	+	-	+
D-Turanose	+	-	+	+	+	-	-	+	+	+	+	-	+
Stachyose	+	-	+	+	+	-	+	+	+	+	+	-	+
D-Raffinose	-	-	-	-	+	-	+	+	-	+	+	-	+
α-D-Lactose	-	-	+	+	-	-	+	+	-	+	+	-	+
D-Melibiose	-	-	+	+	+	-	+	+	-	+	+	-	+
β-Methyl-D-Glucoside	-	-	+	+	+	-	+	+	+	+	+	-	+
D-Salicin	-	-	+	+	+	-	+	+	+	+	+	-	+
N-Acetyl-D-Glucosamine	+	+	+	+	+	+	+	+	+	+	+	-	+
N-Acetyl-β-D-Mannosamine	+	+	+	+	+	-	+	+	+	+	+	-	+
N-Acetyl-D-Galactosamine	+	-	+	+	+	-	+	+	+	+	+	-	+
N-Acetyl Neuraminic Acid	-	-	+	+	+	-	+	+	+	+	+	-	+
α-D-Glucose	+	+	+	+	+	-	+	+	+	+	+	+	+

D-Mannose	+	+	+	+	+	+	+	+	-	+	+	-	+
D-Fructose	+	+	+	+	+	+	+	+	+	+	+	+	+
D-Galactose	+	+	+	+	+	+	+	+	+	+	+	+	+
3-Methyl Glucose	+	-	+	+	+	-	+	+	+	+	+	-	+
D-Fucose	+	+	+	+	+	+	+	+	+	+	+	+	+
L-Fucose	+	+	+	+	+	+	+	+	+	+	+	+	+
L-Rhamnose	+	+	+	+	+	+	+	+	+	+	+	-	+
Inosine	+	+	+	+	+	+	+	+	+	+	+	-	+
D-Sorbitol	+	-	+	-	+	-	+	+	-	+	+	-	+
D-Mannitol	+	+	+	+	+	+	+	+	-	+	+	-	+
D-Arabitol	+	+	+	+	+	+	+	+	+	+	+	-	+
myo-Inositol	+	-	+	+	+	-	+	+	+	+	+	-	+
Glycerol	+	+	+	+	+	+	+	+	+	+	+	+	+
D-Glucose-6-PO4	+	-	+	+	+	-	+	+	+	+	+	-	+
D-Fructose-6-PO4	+	+	+	+	+	+	+	+	+	+	+	+	+
D-Aspartic Acid	+	-	+	+	+	-	+	+	+	+	+	-	+
D-Serine	+	+	+	+	+	+	+	+	+	+	+	+	+
Gelatin	+	+	+	+	+	-	+	+	-	+	+	-	+
Glycyl-L-Proline	+	+	+	+	+	+	+	+	+	+	+	-	+
L-Alanine	+	+	+	+	+	+	+	+	+	+	+	+	+
L-Arginine	+	+	+	+	+	+	+	+	+	+	+	+	+
L-Aspartic Acid	+	+	+	+	+	+	+	+	+	+	+	+	+
L-Glutamic Acid	+	+	+	+	+	+	+	+	+	+	+	+	+
L-Histidine	+	+	+	+	+	+	+	+	+	+	+	+	+
L-Pyroglutamic Acid	+	+	+	+	+	+	+	+	+	+	+	+	+
L-Serine	+	+	+	+	+	+	+	+	+	+	+	+	+
Pectin	+	+	+	+	+	+	+	+	+	+	+	-	+
D-Galacturonic Acid	+	+	+	+	+	+	+	+	+	+	+	+	+
L-Galactonic Acid Lactone	+	+	+	+	+	+	+	+	+	+	+	+	+
D-Gluconic Acid	+	+	+	+	+	+	+	+	+	+	+	+	+
D-Glucuronic Acid	+	+	+	+	+	+	+	+	+	+	+	+	+
Glucuronamide	+	+	+	+	+	+	+	+	+	+	+	+	+
Mucic Acid	+	+	+	+	+	-	+	+	+	+	+	+	+
Quinic Acid	+	+	+	+	+	+	+	+	+	+	+	+	+
D-Saccharic Acid	+	-	+	+	+	-	+	+	+	+	+	+	+
p-Hydroxy-Phenylacetic Acid	+	+	+	+	+	+	+	-	-	+	+	-	+
Methyl Pyruvate	+	+	+	+	+	+	+	+	+	+	+	+	+
D-Lactic Acid Methyl Ester	+	+	+	+	+	-	+	+	+	+	+	-	+
L-Lactic Acid	+	+	+	+	+	+	+	+	+	+	+	+	+

Citric Acid	+	+	+	+	+	+	+	+	+	+	+	+	+
α-Keto-Glutaric acid	+	+	+	+	+	+	+	+	+	+	+	+	+
D-Malic Acid	+	+	+	+	+	-	+	+	+	+	+	+	+
L-Malic Acid	+	+	+	+	+	+	+	+	+	+	+	+	+
Bromo-Succinic Acid	+	+	+	+	+	+	+	+	+	+	+	+	+
Tween 40	+	+	+	+	+	+	+	-	+	+	+	+	+
γ-Amino-Butyric Acid	+	+	+	+	+	+	+	+	+	+	+	+	+
α-HydroxyButyric Acid	+	+	+	+	+	+	+	+	-	+	+	-	+
β-Hydroxy-D,L-Butyric Acid	+	+	+	+	+	+	+	+	+	+	+	+	+
α-Keto-Butyric Acid	+	-	+	+	+	-	+	+	+	+	+	-	+
Acetoacetic Acid	+	+	+	+	+	+	+	+	+	+	+	+	+
Propionic Acid	+	+	+	+	+	+	+	+	+	+	+	+	+
Acetic Acid	+	+	+	+	+	+	+	+	+	+	+	+	+
Formic Acid	+	+	+	+	+	+	+	+	+	+	+	-	+
pH 6	+	+	+	+	+	+	+	+	+	+	+	+	+
pH 5	-	+	+	+	+	+	-	+	-	-	-	-	-
1% NaCl	+	+	+	+	+	+	+	+	+	+	+	+	+
4% NaCl	+	+	+	+	+	+	-	+	-	+	+	-	+
8% NaCl	-	-	-	-	-	-	-	+	-	-	-	-	-
1% Sodium Lactate	+	+	+	+	+	+	+	+	+	+	+	+	+
Fusidic Acid	+	+	+	+	+	+	+	+	-	-	-	-	-
D-Serine.1	-	-	-	-	-	-	-	+	-	-	+	-	-
Troleandomycin	+	+	+	+	+	+	+	+	+	+	+	+	+
Rifamycin SV	+	+	+	+	+	+	+	+	+	+	+	+	+
Minocycline	+	+	+	+	+	+	+	+	-	-	-	-	-
Lincomycin	+	+	+	+	+	+	+	+	+	+	+	+	+
Guanidine HCl	+	+	+	+	+	+	+	+	+	+	+	+	+
Niaproof 4	+	+	+	+	+	+	-	+	+	+	+	+	+
Vancomycin	+	+	+	+	+	+	+	+	+	+	+	+	+
Tetrazolium Violet	+	+	+	+	+	+	+	+	+	+	+	+	+
Tetrazolium Blue	+	+	+	+	+	+	+	+	+	+	+	+	+
Nalidixic Acid	+	+	+	+	+	+	+	+	-	-	-	-	-
Lithium Chloride	-	-	-	-	-	-	-	+	+	+	+	+	+
Potassium Tellurite	+	+	+	+	+	+	+	+	-	+	+	-	+
Aztreonam	-	+	-	-	-	-	-	+	-	-	+	-	+
Sodium Butyrate	-	-	-	-	-	-	-	+	-	-	-	-	-
Sodium Bromate	-	-	-	-	-	-	-	+	-	-	-	-	-

(b)

	NS	NS	NS	NS	NS	NS	NS	CT	CT	CT1	CT1	CT1	CT1
	1	2	3	4	5	6	8	7	9	0	1	2	4
Dextrin	-	-	+	-	-	-	-	-	-	-	-	-	-
D-Maltose	-	-	+	-	-	-	-	-	+	+	+	+	+
D-Trehalose	-	-	+	+	+	-	+	-	-	-	-	-	-
D-Cellobiose	-	-	+	-	+	-	-	-	-	-	-	-	-
Gentiobiose	-	-	+	-	-	-	-	-	-	-	-	-	-
Sucrose	-	-	+	-	+	-	-	-	-	-	-	-	-
D-Turanose	-	-	+	-	+	+	-	-	-	-	-	-	-
Stachyose	-	-	+	-	+	-	-	-	-	-	-	-	-
D-Raffinose	-	-	+	-	-	-	+	-	-	-	-	-	-
α-D-Lactose	-	-	+	-	-	-	-	-	-	-	-	-	-
D-Melibiose	+	-	+	+	-	+	-	-	+	-	-	+	-
β-Methyl-D-Glucoside	-	-	+	+	+	+	-	-	+	-	-	-	-
D-Salicin	-	-	+	+	+	+	-	-	+	-	+	-	-
N-Acetyl-D-Glucosamine	+	+	+	+	+	+	+	-	+	+	-	-	-
N-Acetyl-β-D-Mannosamine	-	+	+	+	+	+	-	-	+	+	+	-	-
N-Acetyl-D-Galactosamine	-	-	+	+	+	+	-	-	+	+	-	-	-
N-Acetyl Neuraminic Acid	-	-	+	+	+	+	-	-	+	-	-	-	-
α-D-Glucose	+	+	+	+	+	+	+	+	+	+	+	+	+
D-Mannose	+	-	+	+	+	+	+	-	+	-	-	-	-
D-Fructose	+	+	+	+	+	+	+	+	+	+	+	+	+
D-Galactose	+	+	+	+	+	+	+	+	+	+	+	+	-
3-Methyl Glucose	+	-	+	+	+	+	+	+	+	+	+	+	-
D-Fucose	+	+	+	+	+	+	+	+	+	+	+	+	+
L-Fucose	+	+	+	+	+	+	+	+	+	+	+	+	+
L-Rhamnose	-	+	+	+	+	+	+	+	+	+	+	+	-
Inosine	+	+	+	+	+	+	+	-	+	+	-	-	-
D-Sorbitol	+	-	+	+	+	+	+	-	-	-	-	-	-
D-Mannitol	+	+	+	+	+	+	+	-	+	+	-	+	-
D-Arabitol	+	+	+	+	+	+	+	+	+	+	+	+	-
myo-Inositol	+	-	+	+	+	+	+	-	+	+	+	+	-
Glycerol	+	+	+	+	+	+	+	+	+	+	+	+	-
D-Glucose-6-PO4	+	-	+	+	+	+	+	-	+	+	+	+	-
D-Fructose-6-PO4	+	+	+	+	+	+	+	+	+	+	+	+	+
D-Aspartic Acid	-	+	+	+	+	+	+	-	+	+	+	+	-
D-Serine	-	+	+	+	+	+	+	+	+	+	+	+	-
Gelatin	+	+	+	+	+	+	+	-	-	-	-	-	-

Glycyl-L-Proline	+	+	+	+	+	+	+	+	+	+	+	+	-
L-Alanine	+	+	+	+	+	+	+	+	+	+	+	+	+
L-Arginine	+	+	+	+	+	+	+	+	+	+	+	+	+
L-Aspartic Acid	+	+	+	+	+	+	+	+	+	+	+	+	+
L-Glutamic Acid	+	+	+	+	+	+	+	+	+	+	+	+	+
L-Histidine	+	+	+	+	+	+	+	+	+	+	+	+	+
L-Pyroglutamic Acid	+	+	+	+	+	+	+	+	+	+	+	+	-
L-Serine	+	+	+	+	+	+	+	+	+	+	+	+	+
Pectin	+	-	+	+	+	+	+	-	+	+	-	-	-
D-Galacturonic Acid	+	+	+	+	+	+	+	+	+	+	+	+	+
L-Galactonic Acid Lactone	+	+	+	+	+	+	+	+	+	+	+	+	+
D-Gluconic Acid	+	+	+	+	+	+	+	+	+	+	+	+	+
D-Glucuronic Acid	+	+	+	+	+	+	+	+	+	+	+	+	+
Glucuronamide	+	+	+	+	+	+	+	+	+	+	+	+	+
Mucic Acid	-	+	+	+	+	+	+	+	+	+	+	+	+
Quinic Acid	+	+	+	+	+	+	+	+	+	+	+	+	+
D-Saccharic Acid	-	-	+	+	+	+	+	+	+	+	+	+	+
p-Hydroxy-Phenylacetic Acid	+	+	+	+	+	+	+	+	-	-	-	-	-
Methyl Pyruvate	+	+	+	+	+	+	+	+	+	+	+	+	+
D-Lactic Acid Methyl Ester	-	-	+	+	+	+	+	+	+	+	+	+	-
L-Lactic Acid	+	+	+	+	+	+	+	+	+	+	+	+	+
Citric Acid	+	+	+	+	+	+	+	+	+	+	+	+	+
α-Keto-Glutaric acid	+	+	+	+	+	+	+	+	+	+	+	+	+
D-Malic Acid	-	-	+	+	+	+	+	+	+	+	+	+	+
L-Malic Acid	+	+	+	+	+	+	+	+	+	+	+	+	+
Bromo-Succinic Acid	+	+	+	+	+	+	+	+	+	+	+	+	+
Tween 40	+	+	+	+	+	+	+	+	+	+	+	+	+
γ-Amino-Butryric Acid	+	+	+	+	+	+	+	+	+	+	+	+	+
α-HydroxyButyric Acid	+	+	+	+	+	+	+	+	+	+	+	+	+
β-Hydroxy-D,L-Butyric Acid	+	+	+	+	+	+	+	+	+	+	+	+	+
α-Keto-Butyric Acid	-	+	+	+	+	+	+	+	+	+	+	+	+
Acetoacetic Acid	-	+	+	+	+	+	+	+	+	+	+	+	+
Propionic Acid	+	+	+	+	+	+	+	+	+	+	+	+	+
Acetic Acid	+	+	+	+	+	+	+	+	+	+	+	+	+
Formic Acid	-	+	+	+	+	+	+	+	+	+	+	+	+
pH 6	+	+	+	+	+	+	+	+	+	+	-	-	-
pH 5	+	+	+	+	+	+	+	-	-	-	-	-	-
1% NaCl	+	+	+	+	+	+	+	+	+	+	+	+	+

4% NaCl	+	+	+	+	+	+	+	-	+	+	+	+	+
8% NaCl	-	+	-	+	-	-	-	-	+	+	+	-	-
1% Sodium Lactate	+	+	+	+	+	+	+	+	+	+	+	+	+
Fusidic Acid	+	+	+	+	+	+	+	-	+	+	+	-	-
D-Serine.1	-	+	-	-	-	-	-	-	-	-	-	-	-
Troleandomycin	+	+	+	+	+	+	+	+	+	+	+	+	+
Rifamycin SV	+	+	+	+	+	+	+	+	+	+	+	+	+
Minocycline	+	+	+	+	+	+	+	-	-	+	+	+	+
Lincomycin	+	+	+	+	+	+	+	+	+	+	+	+	+
Guanidine HCl	+	+	+	+	+	+	+	+	+	+	+	+	+
Niaproof 4	+	+	+	+	+	+	+	+	+	+	+	+	+
Vancomycin	+	+	+	+	+	+	+	+	+	+	+	+	+
Tetrazolium Violet	+	+	+	+	+	+	+	+	+	+	+	+	+
Tetrazolium Blue	+	+	+	+	+	+	+	+	+	+	+	+	+
Nalidixic Acid	+	+	+	+	+	+	+	-	-	+	+	+	+
Lithium Chloride	-	+	-	-	-	-	-	-	+	+	+	+	+
Potassium Tellurite	+	+	+	+	+	+	+	+	+	+	+	+	+
Aztreonam	+	+	+	+	+	+	+	-	-	-	-	-	-
Sodium Butyrate	+	+	+	+	+	+	+	-	-	-	-	-	-
Sodium Bromate	-	-	-	-	-	-	-	-	-	-	+	-	-
